# Impact of type 2 diabetes variants identified through genome-wide association studies in early-onset type 2 diabetes from South Indian population

**DOI:** 10.5808/GI.2020.18.3.e27

**Published:** 2020-09-09

**Authors:** Samuel Liju, Manickam Chidambaram, Viswanathan Mohan, Venkatesan Radha

**Affiliations:** 1 Department of Molecular Genetics, Madras Diabetes Research Foundation, Chennai 600086, India; 2 Dr. Mohan’s Diabetes Specialties Centre, ICMR Centre for Diabetes Advanced Research and WHO Collaborating Centre for Non-communicable Diseases Prevention and Control, Chennai 600086, India

**Keywords:** early-onset type 2 diabetes, genome-wide association studies, *HHEX* gene, MassARRAY genotyping, South Asians, *TCF7L2* gene

## Abstract

The prevalence of early-onset type 2 diabetes (EOT2D) is increasing in Asian countries. Genome-wide association studies performed in European and various other populations have identified associations of numerous variants with type 2 diabetes in adults. However, the genetic component of EOT2D which is still unexplored could have similarities with late-onset type 2 diabetes. Here in the present study we aim to identify the association of variants with EOT2D in South Indian population. Twenty-five variants from 18 gene loci were genotyped in 1,188 EOT2D and 1,183 normal glucose tolerant subjects using the MassARRAY technology. We confirm the association of the *HHEX* variant rs1111875 with EOT2D in this South Indian population and also the association of *CDKN2A/2B* (rs7020996) and *TCF7L2* (rs4506565) with EOT2D. Logistic regression analyses of the *TCF7L2* variant rs4506565(A/T), showed that the heterozygous and homozygous carriers for allele ‘T’ have odds ratios of 1.47 (95% confidence interval [CI], 1.17 to 1.83; p = 0.001) and 1.65 (95% CI, 1.18 to 2.28; p = 0.006) respectively, relative to AA homozygote. For the *HHEX* variant rs1111875 (T/C), heterozygous and homozygous carriers for allele ‘C’ have odds ratios of 1.13 (95% CI, 0.91 to 1.42; p = 0.27) and 1.58 (95% CI, 1.17 to 2.12; p = 0.003) respectively, relative to the TT homozygote. For *CDKN2A/2B* variant rs7020996, the heterozygous and homozygous carriers of allele ‘C’ were protective with odds ratios of 0.65 (95% CI, 0.51 to 0.83; p = 0.0004) and 0.62 (95% CI, 0.27 to 1.39; p = 0.24) respectively, relative to TT homozygote. This is the first study to report on the association of *HHEX* variant rs1111875 with EOT2D in this population.

## Introduction

Early-onset type 2 diabetes (EOT2D) a relatively new phenomenon recognized in the past few decades and is caused by the complex interplay between genetic and environmental factors [1,2]. Currently 425 million people are living with diabetes worldwide and the number is expected to reach 629 million by 2045 with nearly 60% of the affected people living in Asian countries [3,4]. Asians have an earlier age of diagnosis and a higher prevalence of diabetes for the same body mass index (BMI) than Europeans. India alone is presently home to 72 million people with diabetes [4,5]. Epidemiological studies performed in Indians showed a 25.3% increase in individuals developing type 2 diabetes (T2D) at <40 years [6]. A recent nationwide population-based study estimating the national prevalence of diabetes and prediabetes in India also indicates 25–34 years as the take-off point for diabetes both in urban and rural areas [7].

Genome-wide association studies (GWAS) and subsequent meta-analyses of these studies have increased the list of T2D associated genetic variants to more than a hundred [8]. However, these variants identified by the large scale GWAS were mostly with the late-onset type 2 diabetes (LOT2D) subtype that develops after 40 years of age. Though the T2D subtype that develops at earlier ages (EOT2D) has a considerably larger heritable component, very few studies have looked at the genetic component of EOT2D. T2D develops at an earlier age a decade or two earlier in Asian Indians and often coincides with the monogenic form of diabetes namely maturity-onset diabetes of the young (MODY) [9,10]. Indeed previous studies have demonstrated association of some MODY variants also with EOT2D [11-13]. Genetic variants in *TCF7L2* [14-16], *HNF1A* [17], *ABCA1* [18], *DIO2* [19], *PCLO* [20], *TRIB3* [21], *ADIPOQ*, and *LEPR* [22] identified with LOT2D in various populations also showed association with EOT2D. Our group also replicated the association of the variants in *TCF7L2*, *CDKN2A/2B* and an intergenic single nucleotide polymorphism (SNP) on chromosome 1p31 identified with LOT2D in various population also with EOT2D in Asian Indians [23]. With this background, the present study was designed to study the association of 25 variants within 18 distinct gene loci, previously identified with the LOT2D subtype in various GWAS, on South Indians with EOT2D.

## Methods

### Study subjects

The study group comprised of 1,188 unrelated EOT2D subjects and 1,183 normal glucose tolerant (NGT) subjects recruited from Chennai Urban Rural Epidemiology Study (CURES) and from Dr. Mohan’s Diabetes Specialties Centre (DMDSC) tertiary diabetes center in Chennai in South India. Subjects for the study were selected based on the World Health Organization (WHO) criteria. NGT was defined as fasting plasma glucose < 100 mg/dL and 2-h post glucose value ≤ 140 mg/dL. Diabetes was diagnosed if the fasting plasma glucose was ≥ 126 mg/dL or 2-h post glucose value ≥ 200 mg/dL or if the participant was on drug therapy for diabetes after diagnosis by a physician. The following criteria were used for selection of EOT2D subjects: patients having early-onset diabetes if they were diagnosed before the age of 35 years, responding to oral hypoglycemic agents, fasting C-peptide > 1.0, stimulated C-peptide > 2.0 pmol/mL, and glutamic acid decarboxylase antibodies negative. Only unrelated individuals were included in this study. Subjects with ketoacidosis at diagnosis, exocrine pancreatic disease (fibrocalculous pancreatic diabetes), pregnant women and subjects known to have confirmed maturity-onset diabetes of the young, were excluded from the study. Written consent was obtained from all the individuals participating in the study and the study was approved by the Institutional Ethics Committee of the Madras Diabetes Research Foundation (RHN/Adhoc/19/2011-2012).

### Anthropometric and biochemical measurements

Anthropometric measurements including weight, height, and waist measurements were obtained using standardized techniques. The BMI was calculated using the formula, weight (kg)/(height × height)(m^2^). Blood pressure (BP) was measured with a mercury sphygmomanometer (Diamond Deluxe BP apparatus, Pune, India) from the left arm in a sitting position. Fasting plasma glucose (glucose oxidase-peroxidase method), serum cholesterol (cholesterol oxidase-peroxidase-amidopyrine method), serum triglycerides (glycerol phosphate oxidase-peroxidase-amidopyrine method), and high-density lipoprotein cholesterol (direct method polyethylene glycol–pretreated enzymes) was measured using Hitachi-912 Auto analyzer (Hitachi, Mannheim, Germany). Low-density lipoprotein cholesterol was calculated using the Friedewald formula. Glycated hemoglobin was estimated by high-pressure liquid chromatography using the variant machine (Bio-Rad, Hercules, CA, USA) and the intra- and inter-assay coefficient of variation of glycated hemoglobin was less than 10%.

### SNP selection

Twenty-five variants representing eighteen different gene loci identified in various GWAS studies including *ADAMTS9* [24], *CDC123* [25], *CDKAL1* [26-28], *CDKN2A/2B* [24,29,30], *COBLL1* [31], *GRB14* [32], *HNF1A* [33], *HNF4A* [34], *IGF2BP2* [26,28], *JAZF1* [24,33], *HHEX* [35], *PPARG* [35], *RBMS1* [36], *SLC30A8* [27], *TCF7L2* [37], *THADA* [24], *TP53INP1* [34], and *TSPAN8* [34] were selected for the study.

### Genotyping

Genomic DNA was extracted from peripheral blood leucocytes by proteinase K digestion followed by phenol-chloroform method. Genotyping was done using MassARRAY system (Sequenom, San Diego, CA, USA) following the manufacturer's instructions as published elsewhere [38]. SpectroTYPER software (Sequenom) automatically called the genotypes and only conservative and moderate calls were accepted for the study. Ten percent of the samples genotyped were replicated and discordance rate observed was less than 0.4% for the replicated samples. All the variants genotyped had call rate ranging between 90%–99%.

### Statistical analysis

Hardy-Weinberg equilibrium (HWE) was performed by using Pearson χ^2^ statistics in controls for each variant separately. Logistic regression analysis was performed assuming additive model to determine the association between variants and the risk for EOT2D, with and without adjusting for parametric confounders such as age, sex, and BMI using SPSS version 20.0 (IBM Corp., Armonk, NY, USA). The power of the study was estimated using PS Power and Sample Size program (Vanderbilt University, Nashville, TN, USA) calculations (with type I error probability α = 0.05). Linkage disequilibrium (LD) and haplotype frequencies were estimated using Haploview software (http://www.broad.mit.edu/mpg/haploview/) [39].

### Ethical approval

All procedures performed in studies involving human participants were in accordance with the ethical standards of the institutional ethics committee and with the 1964 Helsinki declaration and its later amendments or comparable ethical standards.

## Results

### Clinical and biochemical parameters of the study subjects

Table 1 summarizes the clinical and biochemical parameters of the subjects studied. Mean age of the EOT2D and NGT subjects were 32 ± 6 and 31 ± 8 (mean ± SD), respectively. The fasting plasma glucose, 2-h post plasma glucose, and glycated hemoglobin were significantly (p < 0.001) higher among the EOT2D subjects when compared with the NGT subjects.

### Comparison of minor allele frequencies of the studied polymorphisms in South Indian population with frequencies from the 1000 Genomes Project populations

Minor allele frequencies (MAF) of the SNPs studied in the present study were compared with the reported frequencies of 1000 Genomes Project (Global, European, and South Asian population), representative of the genetic diversity that exists within various population in the world and is shown in Supplementary Table 1. According to the 1000 Genome Project database, MAF of most of the studied SNPs in the present study was similar to the South Asian population, thus supporting the fact that this study population presented a high South Asian component. Similarly, all the SNPs included for the present study were common in South Asian population with allele frequency (MAF) ≥ 0.05.

### LD estimation

**Fig. 1.**
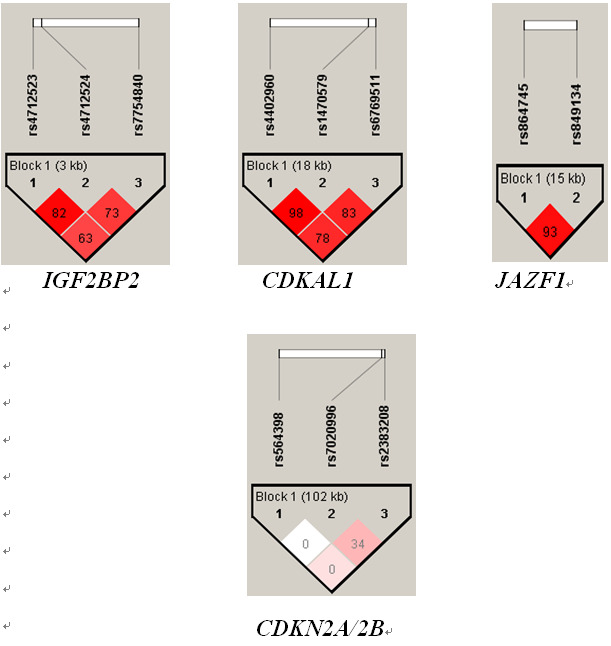
Linkage disequilibrium plot for the single nucleotide polymorphisms (SNPs) of *IGF2BP2, CDKAL1, JAF1*, and *CDKN2A/2B*, genes. R^2^ values mentioned in the linkage disequilibrium (LD) plot. LD is seen only between SNPs of the same gene, not across the genes.

LD analysis was performed for SNPs in *IGF2BP2* (rs4402960, rs1470579, and rs6769511), *CDKAL*1 (rs4712523, rs4712524, and rs7754840), *JAZF1* (rs868745 and rs849134), and *CDKN2A/2B* (rs564398, rs7020996, and rs2383208). Fig. 1 shows the r^2^ values for the studied SNPs. The r^2^ values were found to be at least 0.83 between the SNPs in *IGF2BP2*, *CDKAL*1, and *JAZF1*.

Since the r^2^ values between SNPs in *CDKN2A/2B* was less than 0.35, haplotypes were constructed and the difference in the haplotype frequencies between cases and controls were analyzed. For the SNPs within *CDKN2A/2B*, although the frequency of the TCA haplotype was higher in EOT2D subjects when compared with the NGT subjects (p = 0.027), the significance was lost after Bonferroni correction (p < 0.05/7=0.007). Table 2 shows the haplotype frequencies of the SNPs within *CDKN2A/2B* gene.

### Association of studied SNPs with EOT2D

Genotypic distributions of all the variants studied were in HWE and none of the variants studied showed monoallelic condition. As shown in Table 3, six SNPs within five distinct loci rs7020996 (*CDKN2A/2B*), rs7607980 (*COBLL1*), rs6769511, rs1470579, rs4402960 (*IGF2BP2*), rs4812829 (*HNFA4*), rs1111875 (*HHEX*) and rs4506565 (*TCF7L2*) were found to be significantly associated (p < 0.05) with EOT2D in our South Indian population. However, after Bonferroni correction (p = 0.002) the association with EOT2D remained significant only for three SNPs within three distinct gene loci rs1111875 (*HHEX*: p = 2.0 × 10^-4^), rs4506565 (*TCF7L2*: p = 1.0 × 10^-5^) and rs7020996 (*CDKN2A/2B*: p = 6.0 × 10^-4^). A tendency to association (p < 0.05) with EOT2D was also observed with variants in *COBLL1* (rs7607980), *IGF2BP2* (rs6769511, rs1470579, and rs4402960), and *HNF4A* (rs4812829) in the present study. The risk allele frequencies for all the variants in the EOT2D and NGT subjects are shown in Table 3.

Logistic regression analyses were performed under the additive model for the variants with significant association to EOT2D identified in the present study: rs1111875 (*HHEX*), rs4506565 (*TCF7L2*), and rs7020996 (*CDKN2A/2B*) after adjusting for potential confounders like age, sex, and BMI (Table 4). The heterozygous and homozygous carriers of allele ‘T’ of the *TCF7L2* variant rs4506565 (A/T) had an odds ratio of 1.47 (95% confidence interval [CI], 1.17 to 1.83; p = 0.001) and 1.65 (95% CI, 1.18 to 2.28; p = 0.006) respectively relative to AA homozygote. In the case of the *HHEX* variant rs1111875 (T/C), heterozygous and homozygous carriers for allele ‘C’ had an odds ratio of 1.13 (95% CI, 0.91 to 1.42; p = 0.27) and 1.58 (95% CI, 1.17 to 2.12; p = 0.003) respectively relative to TT homozygote. However, for the *CDKN2A/2B* variant rs7020996 heterozygous carrier for the ‘T’ allele showed an association that was protective in nature with odds ratios of 0.65 (95% CI, 0.51 to 0.83; p = 0.0004) while the homozygous carrier showed no significant association (OR, 0.62; 95% CI, 0.27 to 1.39; p = 0.24) relative to the CC homozygote with EOT2D.

Table 5 shows the comparison of the clinical and biochemical characteristics of NGT subjects and the SNPs associated with EOT2D based on their genotype. For the rs4506565 of the *TCF7L2* gene, NGT subjects homozygous for the ‘TT’ genotype had increased glycated hemoglobin levels (mean ± SD, 5.5 ± 0.4) when compared with the carriers of the ‘AA’ genotype (5.4 ± 0.4, p = 0.01). In case of the *CDKN2A/2B* variant rs7020996, carriers of the ‘CC’ genotype had significantly higher fasting plasma glucose levels (mean ± SD, 87 ± 9 mg/dL), compared to the carriers of the ‘TC’ genotype (85 ± 9 mg/dL, p = 0.03). None of the other biochemical parameters showed any significant differences among the genotypes in either the NGT or the diabetic subjects.

## Discussion

There is a rapid increase in the number of subjects diagnosed with T2D below the age of 40 years. However, only few studies have investigated the association of genetic determinants of LOT2D with EOT2D. The present study aimed at investigating the association of 25 variants from 18 distinct gene loci with EOT2D in this South Indian population, has shown association of variants in *TCF7L2*, *CDKN2A/2B*, and *HHEX* with EOT2D with p-values of 1.00 × 10^-5^, 6.00 × 10^-4^, and 2.00 × 10^-4^ respectively with power ranging from 67%–83%.

Transcription factor-7-like 2 (*TCF7L2*) spans 217kb region on chromosome 10q25.3. *TCF7L2* is a transcription factor involved in the Wnt signaling pathway and is expressed not only in the β-cells but also in other cell lineages and glucose-metabolizing tissues, including the liver [40]. *TCF7L2* identified by Grant et al. [41] is the gene with greater susceptibility to LOT2D in various populations [42-48]. Association of the *TCF7L2* variant rs4506565 (A/T) with LOT2D was initially reported by the Wellcome Trust Case Control Consortium with odds ratio of 1.88 (1.56–2.27, p = 5.1 × 10^-12^) [37]. The association of rs4506565 (*TCF7L2*) with T2D was later was replicated in Middle east [49,50], Tunsanian Arabs [51], Lebanese [52], and Indian population [48,53,54]. While in Europeans, the *TCF7L2* variant rs4506565 showed evidence for association with EOT2D exceeding genome-wide significance, thus clearly establishing *TCF7L2* as a T2D susceptibility gene of substantial importance [42]. Table 6 shows the comparison of the p-value and odds ratio of the SNPs with association to EOT2D identified in the present study with p-value and odds ratio in other population with T2D [24,35,37,49,52,56,56]. The risk allele frequency of the *TCF7L2* (rs4506565) in South Indian EOT2D subjects was observed to be 36.4%, compared with 37% in North Indian subjects [48], 49% in Saudi Arabian subjects [49], 44% in Tunsanian Arab subjects [51], 46% in Lebanese subjects [52] and 39% in European subjects with LOT2D [37]. In the present study, we have shown a strong association of the *TCF7L2* variant with EOT2D in the South Indian population. A previous study by Chidambaram et al. [23] has shown only marginal association of rs4506565 (*TCF7L2*) with EOT2D in Asian Indians. While, the limitation of the previous study was the small sample size, in the present study, we used a much larger sample size thus increasing the power of the study. Rs4506565 (*TCF7L2*) also showed a significant association with fasting glucose in non-diabetic subjects in European population [57]. A comprehensive pathway analysis with 529 of the 548 genes within 5 kb of a *TCF7L2* binding site by Zhao et al. [58] has shown enriched metabolism-related pathway categories in genes bound by *TCF7L2*. Lyssenko et al. [59] using an adenovirus system showed 2-fold increased expression of *TCF7L2* in human islets, associated with increased insulin gene expression and reduced glucose-stimulated insulin secretion compared with control islets. These studies thus provide evidence for increased expression of *TCF7L2* in human islets with altered insulin but not glucagon secretion. *TCF7L2* also plays a crucial role in coordinating the expression of proinsulin and its subsequent processing to form mature insulin [60]. In mouse models, removal of TCF4 from B cells in newborn *Tcf7l2*–/– mice and in adult B cell–specific *Tcf7l2* mutants, challenged by fasting or by high-fat diet did not show any affect in their function [61].

The present study has also confirmed the association of the *CDKN2A/2B* variant rs7020996 with EOT2D in the South Indian population, which was also earlier suggested by Chidambaram et al. [23] in Asian Indian population. The *CDKN2A/2B* locus at chromosome 9p21 was tagged as hot spot for association with LOT2D in a series of GWAS [24,26,30,35]. Zeggini et al. [24] in a meta-analysis study initially reported on the association of rs7020996 of the *CDKN2A/2B* gene with T2D in European population with OR 1.26 (1.15–1.38) (p = 1.8 × 10^-7^). Though *CDKN2A/2B* was reported to influence diabetes risk across varied ethnicities, not many studies have replicated the association of the *CDKN2A/2B* variant rs7020996 with T2D. Replication of the *CDKN2A/2B* variant rs7020996 both with EOT2D and LOT2D by our own group has shown significant association [23,54]. The risk allele frequency of *CDKN2A/2B* (rs7020996) in South Indian EOT2D subjects was observed to be 88.9% in EOT2D subjects, compared with 88% in Asian Indian EOT2D subjects [23] and 91% in South Indian LOT2D subjects [53]. The *CDKN2A/2B* genes are expressed in adipocytes and pancreatic islets. *CDKN2A* and *CDKN2B* encodes p16INK4a and p15INK4b and inhibit the activity of CDK4 and CDK6, respectively. The p16INK4a encoded by *CDKN2A* is a tumor suppressor and inhibits CDK4 (cyclin-dependent kinase) influencing pancreatic β cell proliferation, through decreased cell mass and subsequent decreased insulin release. The increased insulin demand possibly increases the susceptibility to T2D [43]. In murine models, overexpression of Cdkn2a leads to decreased islet proliferation in aging mice and that of Cdkn2b leads to islet hypoplasia and diabetes [62]. Study by Kong et al. [63] investigating the mechanism through which the GWAS identified *CDKN2A/2B* variants increase the T2D risk showed the impact of *CDKN2A/2B* SNPs mediated through β-cell mass but not β-cell function.

*HHEX*, located on chromosome 10q23.33 encodes a 270 amino-acid protein and was identified to be strongly associated with LOT2D in European populations by Scott et al. [35] with odds ratio (OR) 1.13 (1.09–1.17), p = 5.7 × 10^-10^. The association was later replicated in Danish [43], Japanese [55,64,65], Korean [56], Han Chinese [66], and in Tunisian population [67]. However, studies performed in Indians [53,54,68-70] and African American population [71] failed to replicate the association observed in various populations. The lack of association of the *HHEX* variant among various Indian populations Khatri Sikhs [68], Hyderabadi population [69], an endogamous North Indian population [53] and South Indian population [54] could possibly be due to the insufficient sample size, population stratification/admixture or due to confounders. Meta-analysis of 26 studies with 45,792 cases and 65,083 controls, also revealed a stronger association between rs1111875 and risk for T2D in East Asian (OR, 1.19) than in white populations (OR, 1.15) and Indian population (OR, 1.13) [72]. Intriguingly, a meta-analysis by Chauhan et al. [73] performed in Indian population successfully replicated the association of the *HHEX* variant with LOT2D in North Indian population. The risk allele frequency of *HHEX* (rs1111875) in South Indian was observed to be 42.3% in EOT2D subjects, compared with 32% in Japanese [55], 36% in Korean [56], 32% in Han Chinese [66], and 52% in European [35] subjects with T2D. Giannini et al. [74] showed association of the *HHEX* variant rs1111875 with prediabetes among obese youth. In European and Finnish population, the rs1111875 (*HHEX*) showed association with lower birth weight providing evidence for the ‘fetal programming hypothesis’ suggestive of decreased insulin secretion or action with reduced intrauterine growth and thereby lower birth weight as well as susceptibility to LOT2D [75,76]. *HHEX* genes encodes a transcription factor involved in the Wnt signaling pathway and also plays important role in many biological processes including cell cycle regulation, organ development, and cell differentiation via both transcriptional activation and repression [77]. Recent functional studies have identified *HHEX* as the first transcription factor required for δ-cell maintenance mediated through paracrine regulation of β-cell activity. The same study also showed misregulated *HHEX* expression with paracrine control of insulin secretion, leading to accelerated β-cell exhaustion and failure [78]. In *HHEX*-null mice pancreatic β cells was defined for its involvement in β-cell differentiation and function and failure of ventral pancreas development [79]. However, the exact mechanism through which the *TCF7L2*, *CDKN2A/2B*, and *HHEX* exerts its effect on T2D is still unclear. Additionally, a recent study by Mohan et al. [80] has also suggested the predominant role of beta-cell dysfunction than insulin resistance in the pathogenesis of T2D among Asian Indian youth.

With regard to the other variants genotyped in the present study rs6769511, rs1470579, rs4402960 of *IGF2BP2*, rs7607980 of *COBLL1*, and rs4812829 of *HNF4A* showed only a nominal association (p < 0.05) in terms of the association with EOT2D in this South Indian population with power ranging from 34% to 46%. The nominal association of these variants rs6769511, rs1470579, rs4402960 (*IGF2BP2*), rs7607980 (*COBLL1*), and rs4812829 (*HNF4A*) observed with EOT2D in the present study may however be due to the relatively small sample size, which is one of the major limitations of this study. Moreover, we have replicated only 25 gene variants with EOT2D in the present study out of the several hundred gene variants identified with LOT2D.

The significance of EOT2D is that, due to the earlier onset of diabetes these individuals are at increased susceptibility to complications of diabetes includes neuropathy, retinopathy, and cardiovascular disease compared to LOT2D. Our results highlight the need for larger prospective studies to identify the effect of genetic variants implicated in the development of EOT2D. To conclude, the present study is the first study to confirm the association of gene variants associated with EOT2D in South Indian population, and shows the importance of the *HHEX* variants with EOT2D.

## Figures and Tables

**Table 1.** Clinical and biochemical characteristics of the study subjects

Parameter	NGT (n = 1,183)	EOT2D (n = 1,188)	p-value
Age (yr)	31 ± 8	32 ± 6	0.022*
Body mass index (kg/m^2^)	23.5 ± 4.8	26.0 ± 5.4	0.98
Fasting blood sugar (mg/dL)	86 ± 9	177 ± 62	<0.001
2-h post plasma glucose (mg/dL)	99 ± 19	260 ± 85	<0.001
Glycosylated hemoglobin (%)	5.5 ± 0.5	8.9 ± 1.9	<0.001
Total cholesterol (mg/dL)	167 ± 42	173 ± 46	0.002
Log transformed serum triglycerides (mg/dL)	106 ± 65	168 ± 145	<0.001
HDL cholesterol (mg/dL)	44 ± 16	39 ± 11	<0.001

Values are presented as mean ± SD. NGT, normal glucose tolerant; EOT2D, early onset type 2 diabetes; HDL, high-density lipoprotein; SD, standard deviation.

**Table 2.** Haplotype frequencies of SNPs in *CDKN2A/2B*

Haplotype	Cases Freq	Controls Freq	χ^2^	p-value
*CDKN2A/2B* (rs564398, rs7020996, rs2383208)				
TCA	0.621	0.590	4.901	0.027*
CCA	0.216	0.221	0.188	0.664
TTG	0.058	0.072	3.989	0.046
TCG	0.039	0.036	0.318	0.573
TTA	0.024	0.033	3.130	0.769
CTG	0.018	0.020	0.216	0.642
CTA	0.014	0.020	1.952	0.162

SNP, single nucleotide polymorphism.

* p < 0.05, statistically significant.

**Table 3.** The allelic distribution of the variants genotyped in cases and controls

No.	Variant	Gene/nearest gene	Chromosome No.	Risk allele	RAF (%)		p-value
					NGT	EOT2D	
1	rs7607980 (T/C)	*COBLL1*	2	T	91.8	93.7	0.010
2	rs3923113 (A/C)	*GRB14*	2	A	82.6	84.6	0.06
3	rs7593730 (C/T)	*RBMS1*		C	77.2	79	0.14
4	rs7578597 (T/C)	*THADA*		C	89.7	89.3	0.07
5	rs1801282 (C/G)	*PPARG*	3	G	90.1	90.3	0.82
6	rs4607103 (C/T)	*ADAMTS9*		C	46.3	47.5	0.43
7	rs6769511 (T/C)	*IGF2BP2*		C	48.9	52.3	0.03^b^
8	rs1470579 (A/C)			A	47.7	50.9	0.04^b^
9	rs4402960 (G/T)			G	46.7	50.7	0.01^a^
10	rs7754840 (G/C)	*CDKAL1*	6	C	28.2	28.9	0.62
11	rs4712523 (A/G)			G	29.1	30.7	0.24
12	rs4712524 (A/G)			A	26.3	27.8	1.08
13	rs864745 (T/C)	*JAZF1*	7	T	73.9	75.9	0.14
14	rs849134 (A/G)			A	74.2	76.3	0.10
15	rs13266634 (C/T)	*SLC30A8*	8	C	77.5	79.1	0.20
16	rs896854 (C/T)	*TP53INP1*		T	56.4	59.1	0.07
17	rs7020996 (C/T)	*CDKN2A/2B*	9	C	85.5	88.9	6.00 × 10^-4^
18	rs2383208 (A/G)			A	86.8	87.4	0.52
19	rs564398 (T/C)			C	78.7	79.5	0.51
20	rs1111875 (C/T)	*HHEX*	10	T	37	42.5	2.00 × 10^-4^^a^
21	rs4506565 (A/T)	*TCF7L2 *		T	30.1	36.4	1.00 × 10^-5^^a^
22	rs10906115 (A/G)	*CDC123*		G	48.4	50	0.32
23	rs1800574 (C/T)	* HNF1A *	12	C	7.6	8.7	0.17
24	rs4760790 (G/A)	*TSPAN8*		G	36.1	38.5	0.08
25	rs4812829 (A/G)	*HNF4A *	20	A	32.1	35.5	0.008^b^

RAF, risk allele frequency; NGT, normal glucose tolerant; EOT2D, early onset type 2 diabetes.

a p-values with significance threshold after Bonferroni correction (p = 0.05/25 = 0.002).

b Variants showing only borderline significance (p < 0.05);

**Table 4.** Association of variants with early onset type 2 diabetes with OR and CI (adjusted for age, sex, and BMI)

Variant	Genotype	Genotype frequency, n (%)		OR (95% CI) (adjusted for age, sex and BMI)	p-value^a^
		NGT	EOT2D		
rs4506565 (*TCF7L2*)	AA	506 (49.2)	457 (41.3)	Reference	
	TA	412 (40.0)	490 (44.3)	1.47 (1.17–1.83)	0.001
	TT	110 (10.7)	158 (14.3)	1.65 (1.18–2.28)	0.006
rs1111875 (*HHEX*)	TT	438 (40.3)	408 (35.7)	Reference	
	TC	493 (45.4)	505 (44.1)	1.13 (0.91–1.42)	0.27
	CC	156 (14.4)	231 (20.2)	1.58 (1.17–2.12)	0.003
rs7020996 (*CDKN2A/2B*)	CC	805 (73.4)	879 (79.3)	Reference	
	TC	266 (24.2)	214 (19.3)	0.65 (0.51–0.83)	0.0004
	TT	26 (2.4)	15 (1.4)	0.62 (0.27–1.39)	0.24

OR, odds ratio; CI, confidence interval; BMI, body mass index; NGT, normal glucose tolerant; EOT2D, early onset type 2 diabetes.

a p-values were adjusted for age, sex, and BMI.

**Table 5.** Clinical and biochemical characteristics of the NGT subjects stratified based on rs4506565 (A/T) and rs7020996 (C/T) genotypes

Parameter		*TCF7L2*-rs4506565(A/T)			*CDKN2A/2B*-rs7020996 (C/T)			
		AA	AT	TT	CC	TC	TT	
BMI (kg/m^2^)		23.6 ± 4.8	23.7 ± 4.6	23.0 ± 4.7	23.4 ± 4.6	22.9 ± 4.7	23.7 ± 6.2	
Fasting plasma glucose (mg/dL)		86 ± 8	88 ± 9	86 ± 9	87 ± 9^a^	85 ± 9	86 ± 8	
2-h plasma glucose (mg/dL)		98 ± 20	99 ± 19	101 ± 18	99.11 ± 19	98 ± 20	97 ± 18	
Total cholesterol (mg/dL)		170 ± 51	168 ± 33	161 ± 34	168 ± 45	165 ± 33	164 ± 35	
Triglycerides (mg/dL)		106 ± 61	111 ± 79	97 ± 45	104 ± 56^b^	107 ± 83^b^	117 ± 80^b^	
HDL cholesterol (mg/dL)		45 ± 16	45 ± 15	44 ± 17	44 ± 15	44 ± 15	47 ± 28	
LDL cholesterol (mg/dL)		101 ± 30	101 ± 30	96 ± 31	101 ± 30	99 ± 30	93 ± 26	
Glycated hemoglobin (%)		5.4 ± 0.4	5.4 ± 0.4	5.5 ± 0.4^c^	5.4 ± 0.6	5.5 ± 0.5	5.3 ± 0.8	

NGT, normal glucose tolerant; BMI, body mass index; HDL, high-density lipoprotein; LDL, low density lipoprotein.

a p = 0.03 compared to TC (adjusted for age and sex).

b Log transformed values.

c p = 0.01 compared to AA (adjusted for age and sex).

**Table 6.** Comparison of association results for T2D loci in various population with the present study

Gene	Present study		European		Lebanese		Saudi Arabian		Japanese		Korean		
	p-value	OR (95% CI)	European	OR (95% CI)	p-value	OR (95% CI)	p-value	OR (95% CI)	p-value	OR (95% CI)	p-value	OR (95% CI)	
rs4506565	1.13 × 10^-5^	1.33 (1.17–1.51)	5.7 × 10^-12^	1.88 (1.56–2.27) [37]	5.0 × 10^-6^	1.43 (1.20–1.82) [52]	0.007	1.39 (1.09-1.77) [49]	-	-	-	-	
				
	0.0006	1.36 (1.14–.63)	1.8 × 10^-7^		-		-		-		-		
rs7020996				1.26 (1.15–1.38) [24]									
		
rs1111875	0.0002	1.26 (1.11–1.42)	5.7 × 10-^10^	1.13 (1.09–1.17) [35]	-		-		0.0013	1.3 (1.11–1.52) [55]	1.89 × 10^-4^		1.43 (1.18–1.72) [56]

T2D, type 2 diabetes; OR, odds ratio; CI, confidence interval.

## Supplementary Materials

Supplementary Table 1. Comparison of the MAF of the genotyped SNPs with 1000 Genome Project database
