# Supplementary material for: Impact of type 2 diabetes variants identified through genome-wide association studies in early-onset type 2 diabetes from South Indian population
Source: Genomics Inform. 2020 Sep 9;18(3):e27. doi: 10.5808/GI.2020.18.3.e27 (PMC7560451; doi:10.5808/GI.2020.18.3.e27)
Supplement: Supplementary Table 1. — Comparison of the MAF of the genotyped SNPs with 1000 Genome Project database [file gi-2020-18-3-e27-suppl1.pdf]

Supplementary Table 1. Comparison of the MAF of the genotyped SNPs with 1000 Genome Project database

Gene	SNP	Chromosomal location	Minor allele	1000 Genomes MAF			MAF of this study	Functional consequence
				Global	EUR	SAS		
COBLL1	rs7607980	Chr 2:165551201	C	0.107	0.424	0.081	0.063	Missense variant
GRB14	rs3923113	Chr 2:165501849	C	0.385	0.154	0.211	0.154	-
RBMS1	rs7593730	Chr 2:161171454	T	0.247	0.408	0.214	0.210	Intron variant
THADA	rs7578597	Chr 2:43732823	C	0.137	0.090	0.132	0.107	Intron variant
PPARG	rs1801282	Chr 3:12393125	T	0.070	0.793	0.120	0.097	Missense variant
ADAMTS9	rs4607103	Chr 3:64711904	T	0.354	0.120	0.524	0.525	Intron variant
IGF2BP2	rs6769511	Chr 3:185530290	C	0.469	0.232	0.457	0.523	Intron variant
IGF2BP2	rs1470579	Chr 3:185529080	C	0.469	0.304	0.457	0.509	Intron variant
IGF2BP2	rs4402960	Chr 3:185511687	T	0.389	0.304	0.455	0.507	Intron variant
CDKAL1	rs7754840	Chr 6:20661250	C	0.405	0.304	0.256	0.289	Intron variant
CDKAL1	rs4712523	Chr 6:20657564	G	0.419	0.318	0.291	0.307	Intron variant
CDKAL1	rs4712524	Chr 6:20657865	G	0.405	0.319	0.256	0.278	Intron variant
JAZF1	rs864745	Chr 7:28180556	C	0.303	0.318	0.237	0.241	Intron variant
JAZF1	rs849134	Chr 7:28196222	G	0.412	0.519	0.238	0.237	Intron variant
SLC30A8	rs13266634	Chr 8:118184783	T	0.265	0.514	0.255	0.209	Intron variant
TP53INP1	rs896854	Chr 8:95960511	C	0.516	0.283	0.610	0.409	Intron variant
CDKN2A/B	rs7020996	Chr 9:22129579	T	0.258	0.539	0.130	0.111	-
CDKN2A/B	rs2383208	Chr 9:22132076	G	0.210	0.130	0.134	0.126	-
CDKN2A/2B	rs564398	Chr 9:22029547	C	0.185	0.173	0.273	0.205	Intron variant
HHEX	rs1111875	Chr 10:94462882	T	0.456	0.413	0.644	0.425	-
TCF7L2	rs4506565	Chr 10:114756041	T	0.284	0.424	0.304	0.364	Intron variant
CDC123	rs10906115	Chr 10: 12314997	G	0.385	0.345	0.486	0.500	-
HNF1A	rs1800574	Chr 12: 121416864	T	0.020	0.371	0.061	0.087	Missense variant
TSPAN8	rs4760790	Chr 12: 71634794	G	0.236	0.027	0.326	0.385	-
HNF4A	rs4812829	Chr 20:42989267	A	0.255	0.703	0.301	0.358	Intron variant

MAF, minor allele frequency; SNP, single nucleotide polymorphism; EUR, European; SAS, South Asian; Chr, chromosome.
